# Acute Effects of Isometric Contraction Distribution on Jump Performance in Volleyball Players

**DOI:** 10.3390/jfmk10030343

**Published:** 2025-09-09

**Authors:** Jonatan Helbin, Dawid Gawel, Artur Terbalyan, Michal Wilk, Michal Krzysztofik, Danny Lum, Jakub Jarosz

**Affiliations:** 1 Institute of Sport Sciences, Academy of Physical Education, 40-065 Katowice, Poland; jonatan.helbin@gmail.com (J.H.); d.gawel@awf.katowice.pl (D.G.); a.terbalyan@awf.katowice.pl (A.T.); m.wilk@awf.katowice.pl (M.W.); j.jarosz@awf.katowice.pl (J.J.); 2 Department of Sport Games, Faculty of Physical Education and Sport, Charles University, 162 52 Prague, Czech Republic; 3 High Performance Sport Science and Sport Medicine, High Performance Sport Institute, Singapore 397630, Singapore; dannylum82@gmail.com; 4 Sport Performance and Nutrition Research Group, La Trobe University, Melbourne, VIC 3086, Australia

**Keywords:** post-activation performance enhancement, isometric squat, vertical jump, sports performance

## Abstract

**Objectives:** This study evaluated how different distributions of isometric conditioning activity (ICA) durations affect countermovement jump (CMJ) performance in highly trained male volleyball players compared to a control condition (CTRL). **Methods:** Twelve participants performed CTRL and three ICA protocols in a half-back squat: 9 s (3 × 1 × 3 s), 27 s (3 × 3 × 3 s), and 45 s (3 × 5 × 3 s). CMJ height was measured before and at 3, 6, 9, and 12 min post ICA. **Results:** A two-way repeated measures ANOVA showed significant main effects of time (F = 3.820; *p* = 0.009) and condition (F = 6.451; *p* = 0.001), with no significant interaction (F = 1.484; *p* = 0.138). Post hoc analysis indicated significant CMJ height increases at 9 min [mean difference (MD) = 5.1 ± 0.6 cm; *p* = 0.045] and 12 min (MD = 6.0 ± 1.4 cm; *p* = 0.010) post ICA. Moreover, CMJ height was significantly greater in the 27 s (MD = 10.0 ± 0.6 cm; *p* = 0.002) and 45 s (MD = 7.3 ± 2.0 cm; *p* = 0.035) conditions compared to the 9 s protocol. **Conclusions:** Incorporating ICAs of 9 s or 45 s into warm-up routines can enhance CMJ performance, although these durations may elicit different neuromuscular adaptations and movement strategies.

## 1. Introduction

Post-activation performance enhancement (PAPE) refers to a phenomenon whereby specific conditioning activities are combined with post-activation exercise to facilitate short-term improvements in neuromuscular performance [1,2], provided that adequate recovery is given between the two activities [3]. These conditioning activities typically involve high-intensity isotonic or isometric movements conducted prior to a subsequent task requiring similar biomechanical patterns, such as vertical jumps following heavy squats [4,5]. This effect can be explained by its primarily local nature [6,7], which results from the changes that occur in the muscles involved during exercise. Such changes include, among others, phosphorylation of the regulatory light chains of myosin, an increase in muscle temperature, a decrease in pH (leading to the accumulation of H+ ions), enhanced blood flow, an increase in cellular water content, intensification of muscular activity, and an increase in muscle-tendon stiffness [8].

Isometric conditioning activity (ICA) has been attracting growing interest in both scientific research and sports training. However, the findings regarding its efficacy in enhancing acute power performance remain inconsistent [1,5,9,10,11,12,13,14,15,16,17,18]. Recent research on ICAs suggests that one of the key parameters determining the PAPE effect is the volume of ICA, defined as the total duration of isometric contractions (number of sets × duration of each set). Xu et al. [19] proposed that an optimal duration may be approximately 9.6 s. Nevertheless, studies by Spieszny et al. [1] and Bogdanis et al. [20] indicated benefits from employing longer ICA protocols, such as 27 s (3 sets × 9 s). Additionally, the most recent study by Jarosz et al. [15] supported the positive effects of utilizing a longer ICA protocol [27 (3 sets × 9 s) vs. 9 s (3 sets × 3 s)]. This apparent discrepancy suggests that there may not be a single universal optimal duration. Instead, the efficacy of ICA likely depends on the interaction of multiple factors, including the distribution of total contraction time across sets, the type of performance test applied, and the specific training background of the athletes. For instance, in a study by Tsoukos et al. [9], a total ICA duration of 9 s (3 × 3 s) resulted in a significant increase in countermovement jump (CMJ) performance in national-level male track and field power athletes, whereas in studies by French et al. involving track and field athletes [10] and Jarosz et al. involving highly trained male volleyball players [16], the same duration of ICA within a single set did not show a positive response in CMJ. These findings indicate that the concept of an optimal ICA duration should be considered context-dependent, with the interdependence between set structure and contraction time playing a decisive role [16,19]. The underlying mechanisms by which different ICA durations may influence PAPE responses remain insufficiently understood. It is plausible that shorter contractions preferentially enhance neural drive and the recruitment of fast-twitch fibers, whereas longer contractions may additionally induce changes in muscle-tendon stiffness and peripheral fatigue, which could modulate subsequent performance. To date, however, most studies have primarily assessed performance outputs without systematically measuring neuromuscular activity, metabolic responses, or central vs. peripheral mechanisms. This gap highlights the need for further mechanistic investigations (e.g., surface electromyography, near-infrared spectroscopy, or biochemical markers) to clarify how different ICA durations shape physiological responses beyond observed performance outcomes.

Previous studies on the distribution of total ICA duration have primarily focused on dividing contractions into three sets of varying durations: 1 contraction × 3 s = 3 s or 3 contractions × 3 s = 9 s per set, resulting in total ICA durations of 9 or 27 s, which have been shown to induce the PAPE effect [1,9,16,20]. However, there is a lack of studies examining the effects of longer distributions of the total ICA duration. Moreover, the primary energy system during intense efforts in many sports disciplines is the ATP-PCr energy system, which lasts from 0 to 15 s [21]. While isometric contractions do not directly replicate the metabolic demands of dynamic sport actions, considering time domains that partially overlap with these physiological processes may provide useful reference points. For example, the average duration of intense rallies during high-level volleyball matches can also reach up to 15 s [22]. Thus, exploring ICAs of comparable length may be of practical interest from a training perspective, without implying a direct equivalence of energetic profiles. Furthermore, to the best of authors’ knowledge, only two studies have analyzed the impact of 15 s contractions in a single ICA set [10,11], but neither compared different methods of distributing total ICA duration using 3-s contractions in three sets with identical rest periods. Importantly, different methods of distributing isometric contractions (rapid (~1 s), intermittent (~3 s), and sustained (~9 s)) may function differently and influence distinct neuromuscular mechanisms, which may differ from those observed in previous studies [16,22]. As reported in [23], differences in the timing and distribution of contractions may distinctly affect central and peripheral fatigue, as well as motor-unit recruitment strategies. Short, explosive contractions predominantly engage fast-twitch fibers (type II), thereby enhancing neural conduction velocity and force production within ≤100 ms. In contrast, longer, conventional isometric contractions under high load recruit both slow- and fast-twitch fibers, improving neuromuscular control and increasing force output after >200 ms [24]. Such research could contribute to the optimization of training strategies related to the distribution of the total ICA duration, thereby supporting improvements in neuromuscular efficiency, which could positively impact athletic performance outcomes [17]. Moreover, if ICA proves effective, it offers a straightforward implementation that can be easily integrated into various training regimens without the need for specialized equipment [16]. Therefore, this study aimed to address this gap by directly comparing the effects of different methods of distributing the total duration of ICA in comparison to a control condition (CTRL). We hypothesize that the 27 s and 45 s conditions will produce a greater improvement in CMJ height and relative peak power compared to the CTRL conditions due to the potential for more complete neuromuscular synchronization. Additionally, we hypothesize that the conditions with a total ICA duration of 27 s and 45 s will result in a significant increase in CMJ performance at all time points compared to baseline. In contrast to the aforementioned conditions, the 9 s protocol is not expected to significantly affect CMJ height or relative peak power, with results in this case being comparable to the CTRL condition.

## 2. Materials and Methods

### 2.1. Experimental Approach to the Problem

**Figure 1:**
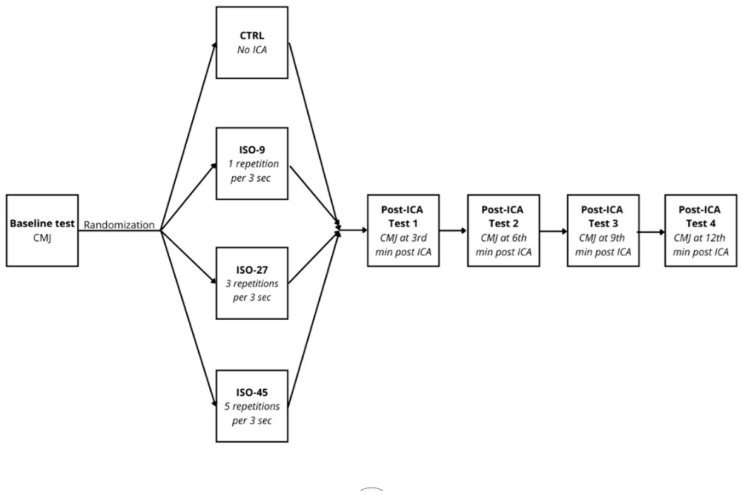
Study design flowchart. CMJ—countermovement jump; ICA—isometric conditioning activity; CTRL—control condition (without ICA); ISO-9—isometric muscle action condition with total ICA duration of 9 s in 3 sets; ISO-27—isometric muscle action condition with total ICA duration of 27 s in 3 sets; ISO-45—isometric muscle action condition with total ICA duration of 45 s in 3 sets.

This investigation employed a randomized crossover design, wherein each participant undertook three experimental trials aimed at examining the immediate influence of maximal isometric squat contractions (utilized as an ICA) on subsequent alterations in CMJ performance. Participants attended a familiarization session and four experimental sessions. During each experimental session, participants performed either one of the three ICA conditions or CTRL. The three conditions were (1) 3 × 1 contraction for 3 s = 9 s (ISO-9), (2) 3 × 3 contractions for 3 s = 27 s (ISO-27), and (3) 3 × 5 contractions for 3 s = 45 s (ISO-45). The CMJ measurements were taken approximately 3 min before CA and 3, 6, 9, and 12 min post ICA [9,17]. In CTRL conditions, measurements were taken at the same time points but without the application of ICA (Figure 1). All sessions were separated by 4–7 days, and all measurements were performed during the in-season.

### 2.2. Participants

The sample size was determined using G*Power version 3.1.9.2 (Dusseldorf, Germany), with the following parameters for the statistical test: “ANOVA for repeated measures with a within-factors” (one group of participants, four experimental conditions, and five measurements), a statistical power of 0.8, a significance level of 0.05, and an effect size of approximately *d* = 0.5 based on previous studies evaluating the immediate impact of isometric activation exercises on jump performance [1,5]. The analysis indicated that the minimum required sample size for this study was 10 participants.

**Figure 2:**
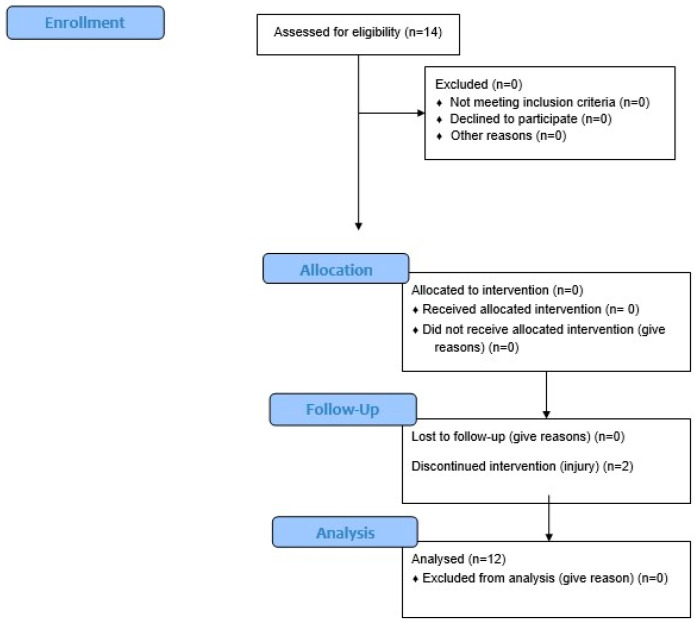
Participant flow diagram.

The study involved 12 highly trained male volleyball players classified based on training status and performance caliber according to McKay et al.’s [25] classification (Table 1). The athletes reported a total training volume of approximately 13 h per week (volleyball practice combined with strength training), which was confirmed by the team’s strength and conditioning coach. The inclusion criteria were as follows: (a) at least 5 years of experience in volleyball training, (b) engagement in a minimum of 3 strength training sessions per week over the last 5 years, and (c) no history of musculoskeletal injuries resulting in a training interruption exceeding 4 weeks for at least 6 months prior to the study commencement. Participants were instructed to adhere to their habitual dietary patterns and to refrain from consuming supplements or stimulants, with the exception of regular supplementation (e.g., creatine) during the week preceding the experiment. Body composition was assessed during the experimental session using multi-frequency bioelectrical impedance analysis under standardized laboratory conditions (InBody 770, Biospace Co., Ltd., Seoul, Republic of Korea). Prior to participation, all individuals received detailed information regarding the potential risks and benefits of the study, as well as their right to withdraw at any time without justification and subsequently provided written informed consent. The study employed a randomized crossover design. While participants were aware of the immediate task they performed (e.g., isometric contractions vs. walking), they remained blinded to the study’s overarching objectives and specific hypotheses. Randomization was carried out using the randomization.org generator, which allocated a unique number and sequence for each participant’s session. After being assigned to a specific study condition, participants were kept unaware of the subsequent stages of the experiment [17]. Detailed information regarding the study’s objectives and anticipated outcomes was also withheld from participants. During the experiment, two participants were excluded due to injuries unrelated to the study. Consequently, the experiment was completed by 12 participants (Figure 2). The entire research protocol was conducted at the Academy of Physical Education in Katowice, Poland. The experiment received ethical approval from the Bioethics Committee for Scientific Research (03/2021) at the Academy of Physical Education in Katowice, in compliance with the ethical guidelines outlined in the 1983 Declaration of Helsinki.

### 2.3. Procedure

Each experimental session began with a standardized warm-up protocol consisting of 5 min of cycling, followed by sets of dynamic exercises: bodyweight squats (10 repetitions), forward lunges (10 repetitions), leg swings (10 repetitions), jumping jacks (10 repetitions), and CMJs (5 repetitions). This warm-up sequence corresponded to the athletes’ habitual pre-training routine [16]. Following the warm-up, participants underwent a baseline assessment of their CMJ performance. After a rest period of approximately 3 min, they performed isometric squats as the ICA, based on the assigned condition, or no ICA (CTRL) in a randomized sequence. During the ICA, a firmly secured immovable barbell was positioned on the participants’ shoulders. The squat depth was standardized at a knee-joint angle of 120° [16,17], with the measurement verified by an experienced coach using a goniometer (EasyAngle, Meloq AB, Stockholm, Sweden). To maintain consistency across trials, body alignment was controlled by a certified weightlifting coach, who ensured a vertical orientation of the torso throughout all experimental conditions. Upon receiving a verbal command from the researcher, participants were instructed to “push the barbell vertically upward as hard and as fast as possible”, pressing their back against the barbell and pushing their feet against the floor [26]. For the CMJ, squat depth was not restricted.

Participants were assigned to one of the following conditions in a random order:

1. CTRL—The control condition, consisting of maintaining light physical activity; participants were to walk on a treadmill at a speed of 5 km/h [1,17] for a time equivalent to performing the entire ICA set (9 min);
2. ISO-9—Three sets of ICA consisting of 1 repetition of 3 s maximal isometric contractions, with a total ICA duration of 1 set per 3 s = 9 s, including 3-min rest intervals between sets;
3. ISO-27—Three sets of ICA, each consisting of 3 repetitions of 3 s maximal isometric contractions, with a total ICA duration of 1 set per 9 s = 27 s, including 3-min rest intervals between sets;
4. ISO-45—Three sets of ICA, each consisting of 5 repetitions of 3 s maximal isometric contractions, with a total ICA duration of 1 set per 15 s = 45 s, including 3-min rest intervals between sets.

### 2.4. Measurement of Countermovement Jump Performance

Jump performance was evaluated utilizing a force platform (Force Decks, Vald Performance, Brisbane, Australia), operating at a sampling frequency of 1000 Hz [27]. This tool is widely recognized for its validity and reliability in assessing the kinematics of vertical jumps [28]. Each participant completed 3 repetitions of CMJ without arm swing, with each attempt separated by 5 s. During testing, participants started from a standing position with their hands placed on their hips, ensuring a neutral posture to minimize hip angular displacement. They were instructed to remain as still as possible for at least one second before initiating the downward phase of the jump. Then, they dropped into the countermovement position to a self-selected depth and, immediately followed by a maximal-effort vertical jump. They were required to land in the same position from which they started, centrally on the force platform. The primary dependent variable was jump height (JH), which was calculated based on the center-of-mass velocity at takeoff using an equation relating to impulse and momentum [17]. Additional variables, including the relative peak power output (PP), modified reactive strength index (RSImod; jump height divided by time to take-off), contraction time (CT; time between the initiation of the countermovement and take-off), countermovement depth (CD; vertical displacement of the center of mass during the preparatory dip), eccentric peak velocity (EPV; maximum downward velocity during the countermovement), and stiffness (S; peak ground reaction force divided by the downward displacement of the center of mass). These factors were considered as potential determinants of jump height.

### 2.5. Statistical Analyses

All statistical analyses were performed using Jamovi (version 2.3.21; The Jamovi Project, Sydney, NSW, Australia). Data visualization was performed using GraphPad Prism version 10.1.1 (GraphPad Software, San Diego, CA, USA). Data are reported as medians with ranges (min–max) or IQR in figures. Normality was assessed via Shapiro–Wilk tests, and because several variables violated the assumption of normality, all inferential statistics were non-parametric. Within-condition (repeated-measures) changes to relative to the reference (0%) over time (3′, 6′, 9′, and 12′) were evaluated using Friedman tests, with effect size indexed by Kendall’s W (small > 0.10, medium > 0.30, large > 0.50). Significant Friedman omnibus effects were followed by Durbin–Conover pairwise comparisons; *p*-values were adjusted for multiple testing with the Holm–Bonferroni procedure to control the family-wise error rate (Type I), and effect sizes for each contrast were reported as rank-biserial correlations (r = Z/√N). Between-group differences at each time point (CTRL, ISO-9, ISO-27, and ISO-45) were tested with Kruskal–Wallis one-way ANOVAs, with epsilon-squared (ε^2^) as the effect-size estimate (small > 0.01, medium > 0.06, large > 0.14). Significant omnibus χ^2^ tests were followed by Dwass–Steel–Critchlow–Fligner pairwise comparisons; likewise, these *p*-values were corrected via the Holm–Bonferroni procedure, and pairwise effect sizes were reported as rank-biserial correlations. Statistical significance was set at *α* = 0.05 throughout.

## 3. Results

A repeated measures two-way ANOVA did not show statistically significant interaction for JH (F = 1.484; *p* = 0.138; ηp^2^ = 0.119) but determined a main effect of time (F = 3.820; *p* = 0.009; ηp^2^ = 0.258) and a main effect of conditions (F = 6.451; *p* = 0.001; ηp^2^ = 0.370) (Table 2).

The post hoc comparisons for the main effect of time showed a statistically significant increase in JH in the 9th and 12th minutes post ICA compared to pre-ICA for JH (mean difference [MD] = 5.1 ± 0.6 cm; Cohen’s d = 0.31; p_bonf = 0.045 for 9th and MD = 6.0 ± 1.4 cm; Cohen’s d = 0.36; p_bonf = 0.010 for 12 min).

The post hoc comparisons for the main effect of condition showed a statistically significant increase in JH in the ISO-9 condition compared to CTRL and the ISO-27 and ISO-45 conditions compared to ISO-9 for JH [MD] = 7.0 ± 0.0 cm, Cohen’s d = 0.34, and p_bonf = 0.047 for ISO-27 compared to CTRL; for MD = 10.0 ± 0.6 cm, Cohen’s d = 0.48, and p_bonf = 0.002 for ISO-27; and for MD = 7.3 ± 2.0 cm, Cohen’s d = 0.36, and p_bonf = 0.035 for ISO-45 compared to ISO-1 (Table 2).

### 3.1. Condition Effects

Significant omnibus effects emerged for ∆% JH at the 3rd min, χ^2^(3) = 9.36, *p* = 0.025, and ε^2^ = 0.199; ∆% PP at the 3rd min, χ^2^(3) = 12.08, *p* = 0.007, and ε^2^ = 0.257; ∆% CT at the 6th min, χ^2^(3) = 7.83, *p* = 0.050, and ε^2^ = 0.167; ∆% RSImod at the 6th min, χ^2^(3) = 7.98, *p* = 0.046, and ε^2^ = 0.170; ∆% CT at the 9th min, χ^2^(3) = 12.36, *p* = 0.006, and ε^2^ = 0.263; and ∆% JH at the 12th min, χ^2^(3) = 8.01, *p* = 0.046, and ε^2^ = 0.170 (Table 3). Post hoc pairwise comparisons, adjusted via Holm–Bonferroni correction within each time-point block, revealed no statistically significant contrasts at α = 0.05. However, nominally significant raw effects were observed for ∆% PP at the 3rd min (ISO-45 vs. ISO-27: W = 4.41, *p* = 0.010; Holm *p* = 0.060), ∆% CT at the 6th min and 9th min (ISO-9 vs. ISO-27: W = −3.76, *p* = 0.040; Holm *p* = 0.240 for each), and ∆% JH at the 12th min (ISO-45 vs. ISO-9: W = −4.08, *p* = 0.020; Holm *p* = 0.120).

### 3.2. Repeated Measures Effects

**Figure 3:**
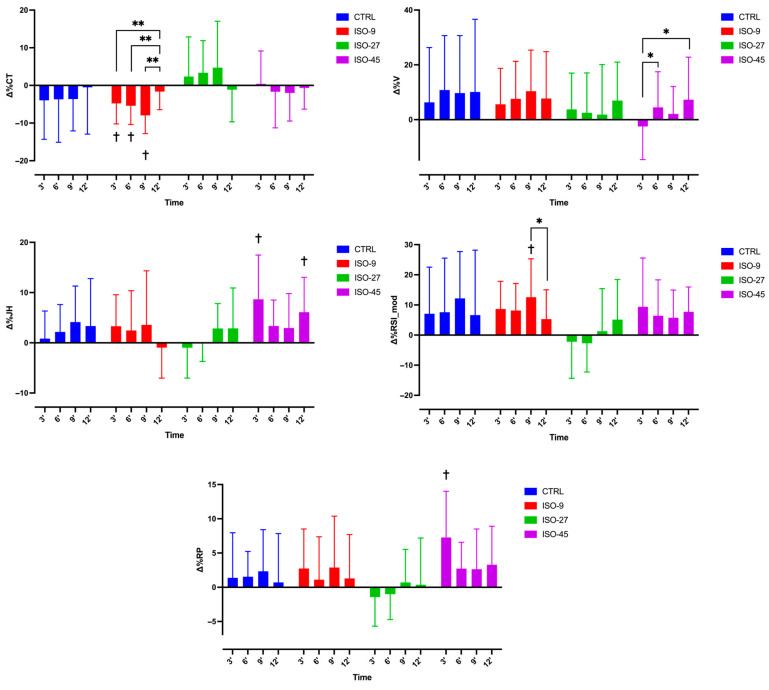
Dynamics of percentage change (Δ%) in CMJ-derived variables across time points (3′, 6′, 9′, and 12′) relative to pre-test (0%) following the ICA or CTRL. Colored bars denote conditions: CTRL (blue), ISO-9 (3 × 1 × 3-s), ISO-27 (3 × 3 × 3-s), or ISO-45 (3 × 5 × 3-s). Statistical significance: * *p* < 0.05; ** *p* < 0.01; † different from reference (*0%). CTRL—control condition; ISO-9—1 repetition of 3 s maximal isometric contractions per set; ISO-27—3 repetitions of 3 s contractions per set; ISO-45—5 repetitions of 3 s contractions per set; JH—jump height; RP—relative peak power output; RSI_mod—modified reactive strength index; CT—contraction time; V—eccentric peak velocity.

In ISO-45, ∆% PP differed across the five time points (χ^2^(4) = 14.90, *p* = 0.01). Post hoc tests with correction showed that ∆% PP at the 3rd min was significantly greater than the reference (*p* = 0.01, r = 1.27); all other pairwise contrasts failed to reach significance after adjustment (all *p* ≥ 0.07; Figure 3).

In ISO-45, ∆% EPV varied significantly across the five measurements (χ^2^(4) = 12.30, *p* = 0.02) (Table 4). Post hoc tests with correction showed that ∆% EPV at the 3rd min differed from the 6th min (*p* = 0.04) and from the 12th min (*p* = 0.04); no other contrasts reached significance after adjustment (all *p* ≥ 0.15; Figure 3).

In the ISO-9 condition, ∆% CT varied significantly across the baseline and the four post-intervention time points (χ^2^(4) = 22.90, *p* < 0.01) (Table 4). Post hoc tests with correction showed that ∆% CT values at the 3rd min (*p* = 0.01), 6th min (*p* = 0.01), and 9th min (*p* = 0.01) each differed from the baseline and that ∆% CT values at the 3rd vs. 12th min (*p* = 0.01), 6th vs. 12th min (*p* = 0.01), and 9th vs. 12th min (*p* = 0.01) also differed significantly. All other pairwise contrasts were non-significant after adjustment (all *p* ≥ 0.18; Figure 3).

In the ISO-9 condition, ∆% RSImod varied across the five measurements (χ^2^(4) = 11.50, *p* = 0.02). Post hoc tests with correction showed that ∆% RSImod at the 9th min exceeded the baseline (*p* = 0.05, r = 0.85); the contrast between the 9th min and 12th min also remained significant (*p* = 0.05, r = 0.67). All other pairwise comparisons did not reach significance after adjustment (all *p*_ ≥ 0.14; Figure 3).

In ISO-45, ∆% JH varied significantly across the five measurements (χ^2^(4) = 10.90, *p* = 0.028). Post hoc tests with correction showed that ∆% JH at the 3rd min was greater than the baseline (*p* = 0.003), and that at 12th min also exceeded baseline (*p* = 0.003); no other time-point contrasts remained significant after correction (all *p* ≥ 0.075; Figure 3).

## 4. Discussion

This study aimed to compare the impact of different distributions of total ICA duration on CMJ performance outcomes in highly trained male volleyball players. The findings showed that the ISO-45 protocol resulted in the greatest improvement in JH at most time points (3rd, 6th, and 12th minutes post ICA) compared to the baseline. However, ISO-27 only resulted in improved JH at the 9th-minute time point, while ISO-9 resulted in improved JH at the 3rd minute and a decrease at the 12th minute. Hence, the current results partially support the hypothesis that both ISO-27 and ISO-45 would result in greater performance improvement than CTRL. Similarly, the hypothesis on ISO-9 not showing a significantly higher increase in JH than CTRL was also partially supported, as both conditions resulted in PAPE at different time points. These results suggest that the distribution of total ICA duration may have a different effect on CMJ performance, and this effect may depend on the measurement time point post ICA and the baseline values of the individual conditions.

To the best of the authors’ knowledge, no previous studies have analyzed the impact of exceptionally long protocols, such as ISO-45, on CMJ performance. In the current study, it was observed that the ISO-45 protocol resulted in the greatest increase in JH and PP at most time points: at the 3rd minute (8.66% ± 8.43% and 7.27% ± 6.46%), 6th minute (3.34% ± 4.95% and 2.73% ± 3.68%), and 12th minute (6.10% ± 6.63% and 3.28% ± 5.40%) post ICA. These findings contradict previous review studies by Xu et al. [19] and Ng et al. [29], who indicated that shorter duration ICAs (total of 9 s) were more effective in inducing PAPE.

A possible explanation for this discrepancy with the current findings could be that the ICA protocols reviewed in those two studies predominantly involved single-joint ICA (e.g., isometric leg extension). It is conceivable that a long-duration, single-joint ICA (total of 10 to 21 s) [29] may have elicited relatively greater fatigue in a specific muscle group, potentially limiting the PAPE response. In contrast, during a multi-joint ICA, the generated forces are distributed across multiple muscles, which may attenuate localized fatigue. Therefore, it is plausible that longer-duration ICA could be more effective in eliciting PAPE when performed as multi-joint isometric actions.

Interestingly, however, despite having to sustain a longer contraction duration for ISO-27 than ISO-9, CMJ performance was not improved during ISO-27, whereas JH and RSImod were improved in ISO-9. This contradicts the hypothesis and does not confirm previous findings of Spieszny et al. [1] and Jarosz et al. [16]. A possible explanation for this could be that participants’ CMJ baseline measures were the highest among all conditions, suggesting that the athletes were closer to their peak performance. Nevertheless, a statistically non-significant improvement was observed at the 9th and 12th minutes post ICA. This suggests that PAPE may take a longer time to manifest when individuals are already close to their peak performance potential, and the magnitude of manifestation may be minimal.

The improvement in JH at the 3rd minute post ISO-9 indicates that a short-duration ICA may also be effective in inducing PAPE. The absence of significant change in JH and PP values in ISO-9 is consistent with the previous findings of Krzysztofik et al. [5]. However, while JH did not improve at other time points, improvement in RSImod was observed at the 12th minute post ICA. The lack of improvement in JH was likely due to the greater reduction in CT up to the 9-min time point. The shorter CT would have resulted in a lower generated impulse—hence, the lack of improvement in JH. However, despite the lower CT to generate a higher impulse, JH was still above the baseline up to the 9th minute. Future studies directly examining the rate of force development are needed to verify whether the higher impulse despite shorter CT reflects an acute enhancement of neuromuscular function. The current results indicate that performing ISO-9 may have altered the neuromuscular response, which led to individuals altering their jump strategy. As team sports require athletes to respond to various situations within a short time (e.g., a volleyball athlete receiving a high-velocity spike), being able to improve on their rate of force development may be a factor for performance. Hence, the ISO-9 condition may also be a viable option for athletes to use during warm-up.

The results should be interpreted with caution, as the observed differences in baseline values across the experimental conditions may have significantly affected the outcomes. These discrepancies may also stem from variations in the body’s responses to changes in experimental conditions or from the athletes’ level of training readiness, which could have been influenced by training load during the season and throughout the study period [30,31,32]. Moreover, although the athletes performed the experimental sessions according to the randomization plan, their training readiness level was not directly monitored. We hypothesize that an analysis of physiological factors, such as biochemical blood tests (including lactate, creatine kinase, and cortisol levels), could have provided additional insights. Another limitation of the present investigation is the lack of non-invasive physiological assessments, which might have yielded a deeper understanding of the mechanisms underlying the observed outcomes. The application of techniques such as surface electromyography or near-infrared spectroscopy would have allowed for a more precise evaluation of neuromuscular activation patterns and metabolic responses without interfering with movement execution, given their ease of implementation and the availability of wireless systems. Incorporating such methodologies could have enhanced the interpretability of the findings and contributed to a more comprehensive understanding of the determinants of the PAPE effect. In addition, the PAPE induced by ICA at different time points was investigated for the same session for each condition. The results may have been altered due to the possible PAPE induced by the preceding CMJ attempts, as plyometric exercises have also been reported to induce PAPE [32]. This may be the reason for the improved JH observed in the CTRL condition at the 9-min time point. Hence, investigating the effect of ICA on PAPE at different time points may best be performed in separate sessions.

## 5. Conclusions

The presented study demonstrates that an ICA of different durations may improve CMJ performance. Our findings suggest that both short- (9 s) and long- (45 s) duration ICA may be considered in warm-up routines, as both durations could potentially enhance lower-limb force generation. However, it should be noted that the two ICA durations may have distinct effects on neuromuscular function and lead to different changes to jump strategies.

## Figures and Tables

**Table 1:** Descriptive characteristics of the study participants.

Age [Years]	27 ± 4
Body mass [kg]	91 ± 13
Body fat [%]	9.9 ± 2.2
Body height [cm]	196 ± 8
Resistance training experience [years]	9.7 ± 4.2
Relative of 1RM BS [kg/bm]	1.48 ± 0.2

1RM—one repetition maximum; BS—back squat; bm—body mass.

**Table 2:** Change in jump height between time points and conditions.

	Group	Baseline	3 Min	6 Min	9 Min	12 Min
Jump Height [cm]	CTRL ISO-9 ISO-27 ISO-45	40.7 ± 4.4 39.9 ± 3.7 42.5 ± 3.7 ^^#^ 40.8 ± 4.3 ^#^	41.0 ± 3.9 41.1 ± 3.6 42.1 ± 4.7 ^^#^ 44.1 ± 4.5 ^#^	41.5 ± 4.0 40.8 ± 3.8 42.4 ± 3.8 ^^#^ 42.0 ± 3.5 ^#^	42.3 ± 4.0 * 41.3 ± 5.4 * 3.7 ± 4.2 *^^#^ 41.8 ± 3.1 *^#^	41.9 ± 4.4 * 41.2 ± 4.8 * 43.7 ± 4.3 *^^#^ 43.1 ± 4.0 *^#^

CTRL—control condition (without ICA); ISO-9—condition with 1 repetition of 3 s maximal isometric contractions within a single ICA set; ISO-27—condition with 3 repetitions of 3 s maximal isometric contractions within a single ICA set; ISO-45—condition with 5 repetitions of 3 s maximal isometric contractions within a single ICA set; *—main effect of time; ^—main effect of conditions in CTRL vs. ISO-45; #—main effect of conditions in ISO-9 vs. ISO-27 and/or ISO-9 vs. ISO-45; —significant difference compared to baseline values at specific time points within the condition, *p* < 0.05.

**Table 3:** Change in jump height, relative peak power, RSImod, and contraction time between time points and conditions.

Variable	Group	3 Min	6 Min	9 Min	12 Min	Time Effect
∆% JH	CTRL	0.41 (−6.04–12.98)	1.27 (−6.12–16.58)	5.25 (−6.54–18.69)	1.21 (−8.07–22.10)	*p* > 0.05
	ISO-9	3.53 (−7.33–12.14)	1.52 (−10.12–20.52)	0.38 (−9.62–22.54)	−1.67 (−7.86–14.95)	*p* > 0.05
	ISO-27	−1.67 (−7.86–14.95)	0.28 (−7.64–5.04)	2.40 (−4.32–10.72)	0.78 (−4.91–19.59)	*p* > 0.05
	ISO-45	8.60 (−6.21–23.51)	3.50 (−5.83–10.59)	3.15 (−6.65–19.46)	4.54 (−5.32–17.67)	χ^2^(4) = 10.90, *p* = 0.028
Condition Effect		χ^2^(3) = 9.36, *p* = 0.025, ε^2^ = 0.199	*p* > 0.05	*p* > 0.05	χ^2^(3) = 8.01, *p* = 0.046, ε^2^ = 0.170	
∆% PP	CTRL	0.66 (−6.92–15.02)	0.49 (−3.29–9.32)	2.17 (−7.34–15.10)	−0.39 (−8.15–13.88)	*p* > 0.05
	ISO-9	1.27 (−6.45–10.83)	−0.26 (−6.94–12.20)	2.03 (−7.92–15.10)	0.62 (−8.29–11.19)	*p* > 0.05
	ISO-27	−2.71 (−5.56–9.01)	−1.18 (−6.45–4.98)	2.04 (−8.42–7.36)	−0.52 (−8.89–12.38)	*p* > 0.05
	ISO-45	4.20 (−3.21–18.23)	2.77 (−3.37–8.18)	2.27 (−5.46–17.20)	2.54 (−6.42–15.07)	χ^2^(4) = 14.90, *p* = 0.01
Condition Effect		χ^2^(3) = 12.08, *p* = 0.007, ε^2^ = 0.257	*p* > 0.05	*p* > 0.05	*p* > 0.05	
∆% RSImod	CTRL	4.75 (−9.80–34.15)	2.22 (−10.94–54.35)	7.83 (−15.62–34.78)	3.65 (−19.61–60.87)	*p* > 0.05
	ISO-9	8.13 (−5.26–23.53)	8.41 (−6.00–27.03)	11.42 (−10.26–29.41)	5.03 (−7.69–23.53)	χ^2^(4) = 11.50, *p* = 0.02
	ISO-27	−3.99 (−22.81–20.46)	−2.78 (−17.86–13.64)	−1.47 (−18.18–34.09)	7.04 (−14.55–26.53)	*p* > 0.05
	ISO-45	12.69 (−20.63–34.69)	8.98 (−25.40–18.61)	4.60 (−4.84–21.95)	6.49 (−5.77–19.51)	*p* > 0.05
Condition Effect		*p* > 0.05	χ^2^(3) = 7.98, *p* = 0.046, ε^2^ = 0.170	*p* > 0.05	*p* > 0.05	
∆% CT	CTRL	−1.13 (−24.41–12.49)	−0.77 (−34.84–6.52)	−2.61 (−25.54–9.54)	1.80 (−36.62–12.73)	*p* > 0.05
	ISO-9	−3.49 (−14.32–3.30)	−5.53 (−11.37–3.11)	−8.67 (−15.84–0.92)	−1.61 (−9.55–5.97)	χ^2^(4) = 22.90, *p* < 0.01
	ISO-27	2.36 (−16.80–27.75)	3.59 (−12.71–15.94)	7.06 (−21.34–20.74)	−1.25 (−16.35–11.80)	*p* > 0.05
	ISO-45	−0.73 (−8.05–22.27)	−4.22 (−12.01–25.21)	−0.74 (−20.39–6.98)	−0.66 (−10.73–10.92)	*p* > 0.05
Condition Effect		*p* > 0.05	χ^2^(3) = 7.83, *p* = 0.050, ε^2^ = 0.167	χ^2^(3) = 12.36, *p* = 0.006, ε^2^ = 0.263	*p* > 0.05	

3 min—3rd minute post ICA; 6 min—6th minute post ICA; 9 min—9th minute post ICA; 12 min—12th minute post ICA; CTRL—control condition (without ICA); ISO-9—condition with 1 repetition of 3 s maximal isometric contractions within a single ICA set; ISO-27—condition with 3 repetitions of 3 s maximal isometric contractions within a single ICA set; ISO-45—condition with 5 repetitions of 3 s maximal isometric contractions within a single ICA set; ∆% JH = percent change in jump height; ∆% PP = percent change in relative power; ∆% RSImod = percent change in modified reactive strength index; ∆% CT = percent change in contact time.

**Table 4:** Change in countermovement depth, eccentric peak velocity, and stiffness between time points and conditions.

Variable	Group	3 Min	6 Min	9 Min	12 Min	Time Effect
∆% CD	CTRL	0.88 (−20.36–15.16)	3.59 (−9.77–11.75)	3.92 (−9.74–13.66)	4.02 (−4.64–24.49)	*p* > 0.05
	ISO-9	−1.97 (−12.61–16.51)	2.12 (−11.28–17.76)	−1.54 (−9.74–19.63)	6.63 (−24.78–22.74)	*p* > 0.05
	ISO-27	4.24 (−10.60–13.37)	2.75 (−10.73–14.38)	1.33 (−10.24–23.63)	4.77 (−20.24–17.27)	*p* > 0.05
	ISO-45	−2.61 (−11.21–8.54)	3.04 (−7.35–8.84)	−0.24 (−12.62–12.81)	3.81 (−7.67–20.33)	*p* > 0.05
∆% EPV	CTRL	−1.84 (−16.78–49.44)	6.27 (−7.69–61.05)	3.24 (−8.76–66.32)	0.00 (−8.80–74.74)	*p* > 0.05
	ISO-9	3.89 (−14.29–29.55)	4.16 (−17.69–39.77)	5.66 (−8.76–40.35)	8.77 (−27.21–35.23)	*p* > 0.05
	ISO-27	2.79 (−24.58–29.57)	3.41 (−17.37–25.51)	−1.51 (−20.37–28.57)	7.85 (−12.57–33.04)	*p* > 0.05
	ISO-45	−1.71 (−32.28–13.99)	6.26 (−29.92–21.24)	3.01 (−17.07–17.36)	4.34 (−12.80–42.98)	χ^2^(4) = 12.30, *p* = 0.02
∆% S	CTRL	−1.53 (−10.58–44.63)	0.69 (−14.23–12.62)	−2.21 (−13.81–13.72)	1.15 (−17.74–19.29)	*p* > 0.05
	ISO-9	6.19 (−13.09–18.15)	1.71 (−18.08–18.27)	7.17 (−6.62–13.57)	−2.01 (−17.23–36.85)	*p* > 0.05
	ISO-27	−1.39 (−18.22–19.92)	−0.94 (−15.43–9.17)	−2.58 (−20.11–16.79)	−1.45 (−17.77–22.78)	*p* > 0.05
	ISO-45	2.80 (−14.48–13.56)	1.55 (−13.89–16.88)	1.99 (−13.18–21.21)	3.21 (−16.54–15.10)	*p* > 0.05

No condition effect for this data was found. 3 min—3rd minute post ICA; 6 min—6th minute post ICA; 9 min—9th minute post ICA; 12 min—12th minute post ICA; CTRL—control condition (without ICA); ISO-9—condition with 1 repetition of 3 s maximal isometric contractions within a single ICA set; ISO-27—condition with 3 repetitions of 3 s maximal isometric contractions within a single ICA set; ISO-45—condition with 5 repetitions of 3 s maximal isometric contractions within a single ICA set; *p* < 0.05. ∆% CD = percent change in countermovement depth; ∆% EPV = percent change in eccentric peak velocity; ∆% S = percent change in stiffness.

## Data Availability

The datasets analyzed during the current study are available from the corresponding author upon reasonable request.

## Abbreviations

The following abbreviations are used in this manuscript:

ICA	Isometric conditioning activity
PAPE	Post-activation performance enhancement
CMJ	Countermovement jump
CTRL	Control condition
ISO-9	Condition with 1 repetition of 3 s maximal isometric contractions within a single ICA set
ISO-27	Condition with 3 repetitions of 3 s maximal isometric contractions within a single ICA set
ISO-45	Condition with 5 repetitions of 3 s maximal isometric contractions within a single ICA set
JH	Jump height
PP	Relative peak power output
RSImod	Modified reactive strength index
CT	Contraction time
CD	Countermovement depth
EPV	Eccentric peak velocity
S	Stiffness
